# Surgical Treatment and Survival in Patients with Liver Metastases from Neuroendocrine Tumors: A Meta-Analysis of Observational Studies

**DOI:** 10.1155/2013/235040

**Published:** 2013-02-20

**Authors:** Stefano Bacchetti, Serena Bertozzi, Ambrogio P. Londero, Alessandro Uzzau, Enrico Maria Pasqual

**Affiliations:** ^1^Department of Surgery, AOU “Santa Maria della Misericordia”, Piazzale SM della Misericordia 15, I-33100 Udine, Italy; ^2^University of Udine and AOU “Santa Maria della Misericordia”, Piazzale SM della Misericordia 15, I-33100 Udine, Italy

## Abstract

*Introduction*. The role of hepatic resection in patients with liver metastases from gastroenteropancreatic neuroendocrine tumors (GEP-NETs) is still poorly defined. Therefore, we examined the results obtained with surgical resection and other locoregional or systemic therapies by reviewing the recent literature on this topic. We performed the meta-analysis for comparing surgical resection of hepatic metastases with other treatments. *Materials and Methods*. In this systematic review and meta-analysis of observational studies, the literature search was undertaken between 1990 and 2012 looking for studies evaluating the different survivals between patients treated with surgical resection of hepatic metastases and with other surgical or nonsurgical therapies. The studies were evaluated for quality, publication bias, and heterogeneity. Pooled hazard ratio (HR) estimates and 95% confidence intervals (CI.95) were calculated using fixed-effects model. *Results*. We selected six studies in the review, five of which were suitable for meta-analysis. We found a significant longer survival in patients treated with hepatic resection than embolisation HR 0.34 (CI.95 0.21–0.55) or all other nonsurgical treatments HR 0.45 (CI.95 0.34–0.60). Only one study compared surgical resection with liver transplantation and meta-analysis was not feasible. *Conclusions*. Our meta-analysis provides evidence supporting the hypothesis that hepatic resection increases overall survival in patients with liver metastases from GEP-NETs. Further randomized clinical trials are needed to confirm these findings and it would be desirable to identify new markers to properly select patients for surgical treatment.

## 1. Introduction

Gastroenteropancreatic neuroendocrine tumors (GEP-NETs) are a heterogeneous group of malignancies with various clinical presentation and growth rates [1–3]. In the current literature, the vast majority of GEP-NETs fall into two nearly distinct categories: pancreatic neuroendocrine tumors, also known as islet cell tumors, and gastrointestinal neuroendocrine tumors, usually grouped in carcinoids [4–6]. In the clinical fashion, gastrointestinal NETs tend to grow much more slowly than pancreatic NETs and also differ in the tumor biology and prognosis [6–8]. It is common to find these tumors in advanced stage, with metastases frequently involving the liver [9–12]. In particular, for gastrointestinal NETs, it is reported that nearly 50–75% of small bowel NETs develop hepatic metastases [13–15]. Although there is uniform consensus for the treatment of primary tumor, there is still debate over how to manage patients with metastatic disease. Many medical and surgical treatments have been proposed for patients with liver metastases from NETs [10, 16–18]. However, the exact role of liver surgery for patients with metastatic NETs is still poorly defined because, frequently, the presence of unresectable hepatic secondaries and the inert growth, and the long-term natural history of the disease make many problems to the evaluation of the real effectiveness of hepatic surgical approach. Moreover, a valid set of criteria for selecting patients to resection has not been established. In the present meta-analysis, our aim was to examine the survival differences of patients treated with surgical resection of hepatic metastases and with other therapies. 

## 2. Materials and Methods

### 2.1. Search Strategy for Review

The literature search was carried out, by three authors independently, by gathering information from Medline, Embase, Ovid, Google Scholar, and Cochrane database for studies published form January 1990 to August 2012. Search terms included “neuroendocrine tumour” or “carcinoid tumour” or “gastrointestinal NETs” or “liver metastases” or “hepatic metastases” or “neuroendocrine metastases” and “hepatectomy” or “liver resection” or “liver transplantation.” Then, we examined all the titles and the abstracts of the resulting articles. The first step was the selection of papers referring to the surgical treatment of liver metastases from NETs. After that, we analyzed the full articles. In addition, bibliographies and citations from full articles and previous review publications were used to identify other additional pertinent articles.

### 2.2. Inclusion and Exclusion Criteria

We considered for inclusion all experimental and observational studies that evaluated survival in patients affected by NET liver metastases and treated by hepatic surgical resection or liver transplantation or other therapies (somatostatin analogues, hepatic embolisation/chemoembolisation, peptide receptor therapy, chemotherapy, etc.) and submitted to watchful waiting. All relevant studies were observational (level III or IV of evidence, CEBM) [25] because randomized trials comparing partial hepatectomy versus liver transplantation or other nonsurgical therapies have never been attempted. In this meta-analysis we considered a Cox proportional hazards regression model or Kaplan-Meier curves to calculate the survival difference among patients treated with resection of liver metastases and other treatments. Moreover, we included only articles written in English on human subjects with the full text available for data retrieval. We recorded geographic locations, time frame for NET diagnosis, and treatment in order to avoid any possible population overlap. When two or more studies presented possible overlap, the one of better quality or with more detailed data was included. In case of discrepancies among the three reviewers they were addressed by a joint reevaluation of the original article. Specific exclusion criteria were studies considering <20 patients, or nonhuman subjects, and non-English written articles. Furthermore, we excluded also reviews, letters to the editor without original data, editorials, and case reports. Conference abstracts were also excluded due to a lack of details regarding the study design and survival data. 

### 2.3. Data Extraction

Three reviewers independently extracted data for selected studies using a standard data extraction form. They discussed any discrepancies in appropriateness for inclusion in the present meta-analysis and data extraction. The following information was then extracted from every single study: authors, year of publication, geographical area, population characteristics (age, sex, etc.), study design, number of patients, type of procedure applied, hazards ratios with 95% confidence interval (CI.95), or hazards ratios extracted from Kaplan-Meier curves. The hazard ratio in our meta-analysis was calculated from data obtained from published reports, using methods previously described [26]. 

### 2.4. Quality Assessment for Included Studies

Three authors assessed independently the quality of each included study by using the Newcastle-Ottawa Scale [27]. The Newcastle-Ottawa Scale evaluates the quality of studies analyzing three items: selection, comparability, and outcome (cohort studies) or exposure (case-control studies). This scale assigns a maximum of nine points to each study: a maximum of four points for selection, two points for comparability, and three points for exposure/outcome. Therefore, the highest quality is achieved by scoring nine points. In our analysis studies of high quality were defined those that scored nine or eight points and studies of medium quality those that scored seven or six points on the Newcastle-Ottawa Scale. Any discrepancy in quality assessment was addressed by a joint evaluation of the original article. 

### 2.5. Data Analysis

The data was analyzed by R (version 2.15.0), considering significant *P* < 0.05. In the meta-analysis, a summary statistic was calculated considering the hazards ratio for survival analysis. We used rank correlation test of funnel plot asymmetry to test the presence of any publication bias [28, 29]. We used I2 index and Cochran Q to assess the heterogeneity among studies. We considered an I2 index value > 50% a measure of heterogeneity and, for Q statistic, a *P* value < 0.10 was considered statistically significant for heterogeneity [30]. The fixed- and random-effect models were applied to calculate the pooled estimate where appropriate. The primary outcome in this meta-analysis was reported as HR (with CI.95) of overall survival in patients treated with hepatic resections. We considered MOOSE (Meta-Analysis Of Observational Studies in Epidemiology) guidelines for accurately performing meta-analysis of observational studies [31] and PRISMA (Preferred Reporting Items for Systematic Reviews and Meta-Analyses) guidelines checklist [32]. 

## 3. Results

### 3.1. Search Results

**Figure 1:**
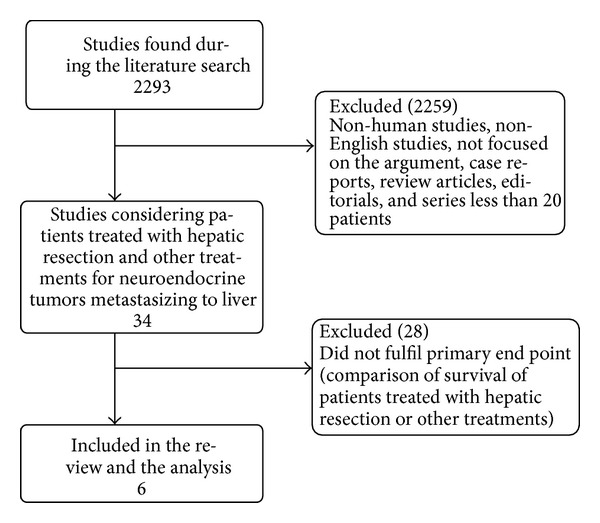
Flow-chart of the literature search and selection.

We identified a total of 2293 articles during the initial search (Figure 1). After reviewing the titles and abstracts of these publications, 2259 were found to be not eligible as they were case reports, review articles, editorials, nonhuman studies, or non-English articles, not focusing on the review topic, and not meeting the inclusion criteria. In total, 34 articles were identified as potentially eligible for this review. According to a subsequent evaluation of full-text articles, 28 of these articles either described only the outcome of patients treated with surgical management or did not report any HR or Kaplan-Meier curves to compare surgical resection of hepatic metastases with other treatments (Supplemental List 1) (see Supplementary Material available online at http://dx.doi.org/10.1155/2013/235040). We finally selected six eligible articles that compared survival between the groups using Kaplan-Meier curves (Figure 1) [19–24]. All included studies were observational retrospective studies. Five studies compared hepatic metastases resection with other conservative treatments and one study compared surgical resection with liver transplantation. 

### 3.2. Characteristics of the Studies

This meta-analysis included retrospective observational studies that evaluated survival in patients affected by NET comparing surgical resection of hepatic metastases with conservative management or other treatments (Table 1) [19–24]. Five studies compared surgical resection with conservative treatments and one study compared surgical resection with liver transplantation. Among the five studies comparing surgical resection with conservative treatments two studies compared surgery to embolisation. In Table 1 we present the characteristics of the included studies. None of the studies presented Cox proportional hazards multivariate regression models and HR was extracted from Kaplan-Meier curves. We could not perform any meta-analysis about surgical resection versus liver transplantation, because there was only one eligible study. In this study, Coppa et al. found a non significant increased OS in the group treated with liver transplantation versus surgical resection of liver metastases alone [24]. In Supplemental List 1 we show also the excluded studies after analyzing the full paper during the second step of our study selection process. All these studies were observational and retrospective and HR extraction to perform the meta-analysis was not possible. The majority of the excluded studies were accurately described and summarized by a recent systematic review by Saxena et al. [33]. 

### 3.3. Quality Assessment of the Included Studies

The quality of the evidence on the influence of surgical treatment on survival of patients with NET liver metastases is quite low (levels III-IV, CEBM) [25]. 

All studies in our meta-analysis consistently showed an increased survival in the groups treated with surgery but none of the studies was randomized. The three independent reviewers agreed that all studies were graded six or seven points on the nine-point Newcastle-Ottawa scale for quality (medium quality). We regarded our results to be the basis to plan randomized clinical trials on this field. 

### 3.4. Main Analysis

**Figure 2:**
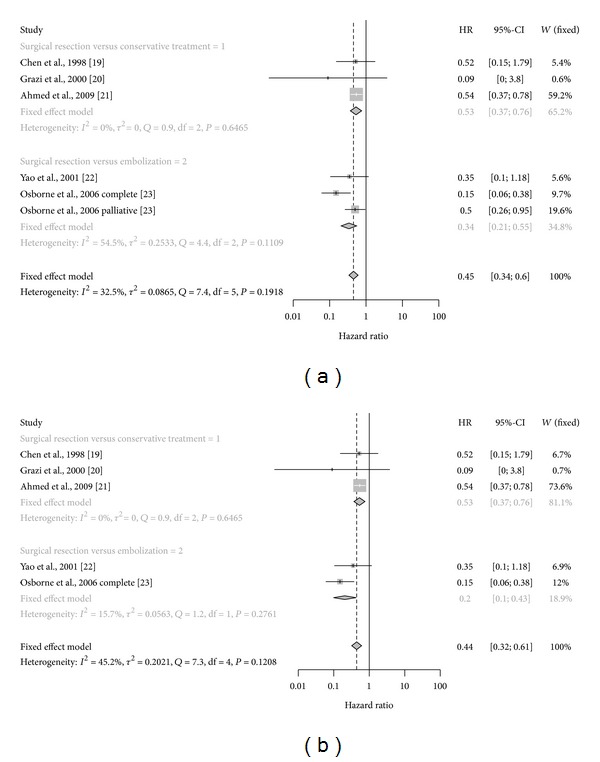
(a) Forest plot of overall survival comparison between hepatic metastasis resection versus expectant or other conservative/minimally invasive managements. (b) Analysis of OS excluding palliative surgery from data published by Osborne et al. [23].

The meta-analysis was performed on five studies as reported in Figure 2 and considered 374 patients affected by NET liver metastases and treated in a conservative manner and 161 patients treated with liver metastases. The heterogeneity among the studies was not significant and we used fixed-effect model to calculate the pooled estimate. Only considering data from incomplete cytoreduction group published by Osborne et al. we found significant heterogeneity but fixed- and random-effect models were similar and for this study we considered the fixed-effect model [23]. We found a significant increased survival in the group of patients treated with surgical hepatic resections HR 0.45 (CI.95 0.34–0.60) in comparison to conservative treatments and to embolization HR 0.34 (CI.95 0.21–0.55) (Figure 2(a)). In Figure 2(b) we reported the same analysis excluding only the cases treated with palliative surgery by Osborne et al. and the results were similar [23]. 

### 3.5. Risk of Bias Assessment

All the observational retrospective studies were classified as medium quality. We did not find any randomized clinical trial on this argument. The main limit to consider observational retrospective studies was to suppose a possible selection bias for the patients treated with surgical resection (limited disease in comparison to the group treated with conservative treatments). The articles of Yao et al. and Chen et al. stated that surgical resection and conservative treatment groups presented comparable disease characteristics [19, 22] and the study of Osborne et al. showed comparable pretreatment status between surgical resection and embolization [23]. The article published by Ahmed et al. presented the widest population and the patients treated in a conservative manner were older, with low proliferative index and high Chromogranin A [21]. The study of Grazi et al. did not specify the differences between the group treated with surgical liver resection of NET metastases and the conservative treatment group [20]. 

### 3.6. Publication Bias

**Figure 3:**
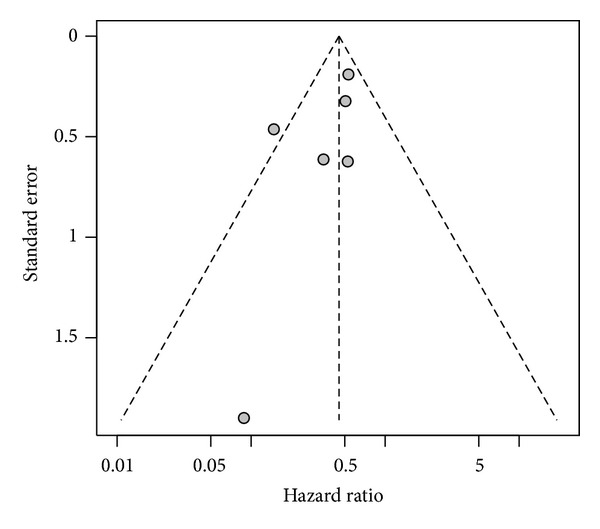
Funnel plot.

We show in Figure 3 a funnel plot examining possible publication bias. These results should be interpreted with caution, because our meta-analysis calculation included only five studies (Figure 2, six dots because one study considered separately complete and palliative cytoreduction), and current guidelines do not recommend testing for funnel plot asymmetry in analysis of a limited number of studies (<10) [34]. The study with the smallest number of controls seems to be out of the symmetry in the plot (Figure 3). Also the comparison between complete cytoreductive surgery and embolization seems out of the symmetry. Nevertheless, the rank correlation test of funnel plot asymmetry with a *P* value of 0.189 does not indicate significant asymmetry in the funnel plot. 

## 4. Discussion

In patients with NETs, occurrence of hepatic secondaries is one of the most important prognostic factors for survival [10, 35–37]. Due to the high prevalence of distant metastases and recurrences, NETs must be considered to have malignant potential [2, 38–41]. In particular, pancreatic NETs showed the lowest 5-year survival rates (34.1–37.6%), whereas gastrointestinal NETs exhibited the highest survival (85.9–88.5% at 5 years) [2, 35, 42]. Even if these neoplasms are quite uncommon, 2% of all malignancies [3], the incidence of NETs has increased exponentially (overall 500%) over the last three decades [3]. So the traditional assumption that these cancers are rare is incorrect [2]. Actually, the medical and surgical therapy of NETs is a hot topic and during the last two years at least four reviews of the literature have been published on this subject [6, 33, 43, 44]. 

The most recent classifications of the 7th American Joint Committee on Cancer/Union International Contre le Cancer 2009 (AJCC/UICC) and of the European Neuroendocrine Tumor Society 2006 (ENETS), associated with the WHO classification 2010, segregate NETs into well-differentiated neuroendocrine tumors (low and intermediate grade based on the Ki67 labeling index, also named NET-G1 or carcinoid and NET-G2, resp.) and into the group of neuroendocrine carcinomas (high grade, poorly differentiated, also named NEC) [40]. These classifications have provided means to better grade and stage NETs, but although these classifications may be useful for primary tumors, they do not allow the stratification of patients with hepatic neuroendocrine metastases [8, 45]. 

Considerable controversy exists regarding the best approach to patients with NET hepatic metastases. The management of these patients varies from control of symptoms only to more aggressive surgical or conservative therapies. For patients with unresectable liver disease, biotherapy with somatostatin analogues, peptide-mediated radioreceptor therapy, transarterial chemoembolisation, selective intra-arterial radiotherapy, or new molecular target-directed therapy can be employed [4, 44, 46], but these therapies are considered as palliative [18]. For localized hepatic metastases, surgical therapy appears as the most efficient approach [5, 16, 17, 36, 37, 47–49]. Furthermore, surgical resection of hepatic metastases could significantly reduce carcinoid symptoms [33]. 

Many studies that evaluate the outcome following surgical management of liver metastases from NETs have focused solely on resection rather than combined-modality approaches that include resection and ablation or surgery and chemotherapy. It is well known that neuroendocrine liver metastases recur in the most patients after hepatic resection, with high recurrence rates up to 70–94% at 5 years [8, 22, 35, 37, 49–51] and the liver is the most common site of progression of disease (69%) [49]. Therefore, the true curative role of liver-directed surgery was questionable and new strategies in association to surgery should be studied. It is important to underline that data on repeated liver surgery for recurrent disease have been extremely limited, and the role of repeated operations remains ill defined [51]. 

In our review and meta-analysis, we found five studies comparing surgical resection of NET liver metastases with conservative management and we observed a significant increased survival in patients treated with liver resection. It is important to underline that all the included studies were observational and so the clinical evidence was low. 

A recent systematic review considering 29 studies (between 1980 and 2009) found a 5-year OS of 70.5% (range 31–100%) and a 5-year progression-free survival of 29% (range 6–66%) [33]. Histological grade, extrahepatic disease, and macroscopically incomplete resection of liver metastases were associated with poor prognosis. Moreover, it was found that the predominant histological type was carcinoid (71%) and the most common origin was the small/large intestine (52%) [33]. In another multi-institutional study evaluating 339 patients, Mayo et al. demonstrated with multivariate analysis that synchronous disease, nonfunctional NET hormonal status, and extrahepatic disease were the most important predictive factors of worse survival [51]. Concerning other prognostic parameters for primary NETs and liver metastases, Katz et al. demonstrated that the robust presence of tumour-infiltrating lymphocytes is a significant predictor of outcome [52]. Moreover, it is demonstrated that Ki67 staining of core biopsies provides an adequately reliable method of proliferation assessment for prognosis of metastatic NETs to the liver [45]. 

The symptomatology is the most important consequence of NETs liver metastases (particularly for carcinoid tumors). Normally serotonin produced by a carcinoid tumor from a primary gut localization is secreted into the portal circulation and metabolized within the liver. The presence of multiple bilobar liver metastases will cause carcinoid syndrome because the serotonin is not metabolized and directly secreted into the systemic circulation. Furthermore, Saxena et al. found that 95% of patients (range 50–100%) will benefit from liver surgical resection by reducing symptomatology. They concluded that hepatic resection was safe and effective in symptomatic relief and favorable survival outcomes although the majority of patients develop recurrence of disease. It was hypothesized that the recognition of new markers could better identify which patients will be selected for surgery [33]. 

As previously stated, we agree that data about the survival benefit must be interpreted with caution because they could likely just to be the product of prudent patient selection. Probably, in the majority of published studies, there was a selection bias because many patients with a large number of hepatic tumors tend to be managed without surgical resection. Likewise, patients with synchronous disease are more likely to be treated without surgery. As randomized controlled trials are not available yet, the question about the effectiveness of surgical resection and other treatment modalities remains unanswered. Anyway, in our meta-analysis, at least two studies considered comparable disease in surgical and nonsurgical groups [19, 22]; in the study of Osborne et al. pre-treatment statuses between surgical resection and embolization were similar, and in the study of Ahmed et al. the difference is unlikely to be due only to patient selection [21]. Even the analysis of the two studies with comparable characteristics was in the same direction of the global analysis, and thus in favour of hepatic resection [19, 22]. Another weakness of our study is related to the great heterogeneity and wide variety in conservative therapies undertaken for NETs, which is due to the observational nature of the included studies. To overcome this weakness we subdivided the analysis considering separately the two studies comparing only surgery and embolization. Also in these two studies we found an advantage of surgical resection over conservative treatment (only embolization in this subgroup) in terms of overall survival. This further analysis resulted in the same direction as the overall analysis thus in favor of surgical resection. Therefore, we found a significant increased survival in the surgical hepatic resection group, and this data requires further evidence from randomized clinical trials in order to obtain a definitive answer to a really important question like the survival gain in these patients. 

Our literature search results included only one study about liver transplant and we could not do any meta-analysis [24]. Recurrence after liver transplantation to treat NETs remains a significant concern, and considering the high morbidity and mortality associated with this procedure the indication of symptom relief alone must outweigh the significant risks [53]. Appropriate selection criteria and further international multicentric studies are needed to demonstrate survival and clinical benefits of this procedure. 

## 5. Conclusion

Liver metastases are frequently encountered in patients with NETs; these secondaries have an important role in the prognosis. The published observational studies and our paper were supporting the surgical solution for NET hepatic metastases. But the observational and retrospective nature of these studies was limiting the level of evidence to support this solution. Since no randomized trial has been published, which could inform meaningfully about the sustained advantages of hepatic resection, no certain conclusion on the impact of this aggressive approach can be achieved. Our meta-analysis based on observational studies found a significant increased survival after surgical hepatic resection, but randomized clinical trials must be undertaken to achieve more evidence about the role of surgical treatment in patients with liver metastases from NETs.

## Supplementary Material

Supplemental List 1- Detailed list of included and excluded studies from studies considering patients treated with hepatic resection and other treatments for neuroendocrine tumors metastatizing to liver.

## Figures and Tables

**Table 1:** Description of the included studies.

Labels	Location (city, country)	Publication year	Study period	Number of patients (liver resection/other)	5 ys OS (liver resection/other)
Surgical resection versus other conservative treatments					

Chen et al., 1998 [19]	Baltimore (USA)	1998	1984–1995	15/23	73%/29%
Grazi et al., 2000 [20]	Bologna (Italy)	2000	1981–1997	19/9	92.6%/18.5%*
Ahmed et al., 2009 [21]	Basingstoke, London, Liverpool, Belfast, and Southampton (UK)	2009	1973–2007	50/269	78%/52%

Surgical resection versus embolization					

Yao et al., 2001 [22]	Chicago (USA)	2001	1992–2000	16/20	70%/40%
Osborne et al., 2006 [23]	Tampa (USA)	2006	2000–2004	38 complete and 23 palliative/53	78% complete and 64% palliative/35%

Surgical resection versus liver transplant					

Coppa et al., 2001 [24]	Milan (Italy)	2001	1987–1999	20/9	67%/70%

*Four years OS.

## Abbreviations

NET: Neuroendocrine tumor
RCT: Randomized controlled trial.
