# Supplementary material for: Surgical Treatment and Survival in Patients with Liver Metastases from Neuroendocrine Tumors: A Meta-Analysis of Observational Studies
Source: Int J Hepatol. 2013 Feb 20;2013:235040. doi: 10.1155/2013/235040 (PMC3590743; doi:10.1155/2013/235040)
Supplement: Supplementary file 1 — Supplemental List 1- Detailed list of included and excluded studies from studies considering patients treated with hepatic resection and other treatments for neuroendocrine tumors metastatizing to liver. [file 235040.f1.doc]

**Supplemental List 1**

**INCLUDED STUDIES**: fulfilled the requirements.

1) Ahmed, A.; Turner, G.; King, B.; Jones, L.; Culliford, D.; McCance, D.; Ardill, J.; Johnston, B. T.; Poston, G.; Rees, M.; Buxton-Thomas, M.; Caplin, M. & Ramage, J. K. (2009), 'Midgut neuroendocrine tumours with liver metastases: results of the UKINETS study.', *Endocr Relat Cancer* **16**(3), 885--894.

2) Chen, H.; Hardacre, J. M.; Uzar, A.; Cameron, J. L. & Choti, M. A. (1998), 'Isolated liver metastases from neuroendocrine tumors: does resection prolong survival?', *J Am Coll Surg* **187**(1), 88--92; discussion 92-3.

3) Coppa, J.; Pulvirenti, A.; Schiavo, M.; Romito, R.; Collini, P.; Di Bartolomeo, M.; Fabbri, A.; Regalia, E. & Mazzaferro, V. (2001), 'Resection versus transplantation for liver metastases from neuroendocrine tumors.', *Transplant Proc* **33**(1-2), 1537--1539.

4) Grazi, G. L.; Cescon, M.; Pierangeli, F.; Ercolani, G.; Gardini, A.; Cavallari, A. & Mazziotti, A. (2000), 'Highly aggressive policy of hepatic resections for neuroendocrine liver metastases.', *Hepatogastroenterology* **47**(32), 481--486.

5) Yao, K. A.; Talamonti, M. S.; Nemcek, A.; Angelos, P.; Chrisman, H.; Skarda, J.; Benson, A. B.; Rao, S. & Joehl, R. J. (2001), 'Indications and results of liver resection and hepatic chemoembolization for metastatic gastrointestinal neuroendocrine tumors.', *Surgery* **130**(4), 677--82; discussion 682-5.

6) Osborne, D. A.; Zervos, E. E.; Strosberg, J.; Strosberg, J.; Boe, B. A.; Malafa, M.; Rosemurgy, A. S.; Yeatman, T. J.; Carey, L.; Duhaine, L. & Kvols, L. K. (2006), 'Improved outcome with cytoreduction versus embolization for symptomatic hepatic metastases of carcinoid and neuroendocrine tumors.', *Ann Surg Oncol* **13**(4), 572--581.

**EXCLUDED STUDIES**: because not focused on the problem, or overlapping data.

1) Chamberlain, R. S.; Canes, D.; Brown, K. T.; Saltz, L.; Jarnagin, W.; Fong, Y. & Blumgart, L. H. (2000), 'Hepatic neuroendocrine metastases: does intervention alter outcomes?', *J Am Coll Surg* **190**(4), 432--445.

2) Chambers, A. J.; Pasieka, J. L.; Dixon, E. & Rorstad, O. (2008), 'The palliative benefit of aggressive surgical intervention for both hepatic and mesenteric metastases from neuroendocrine tumors.', *Surgery* **144**(4), 645--51; discussion 651-3.

3) Cho, C. S.; Labow, D. M.; Tang, L.; Klimstra, D. S.; Loeffler, A. G.; Leverson, G. E.; Fong, Y.; Jarnagin, W. R.; D'Angelica, M. I.; Weber, S. M.; Blumgart, L. H. & Dematteo, R. P. (2008), 'Histologic grade is correlated with outcome after resection of hepatic neuroendocrine neoplasms.', *Cancer* **113**(1), 126--134.

4) Chung, M. H.; Pisegna, J.; Spirt, M.; Giuliano, A. E.; Ye, W.; Ramming, K. P. & Bilchik, A. J. (2001), 'Hepatic cytoreduction followed by a novel long-acting somatostatin analog: a paradigm for intractable neuroendocrine tumors metastatic to the liver.', *Surgery* **130**(6), 954--962.

5) Elias, D.; Lasser, P.; Ducreux, M.; Duvillard, P.; Ouellet, J.-F.; Dromain, C.; Schlumberger, M.; Pocard, M.; Boige, V.; Miquel, C. & Baudin, E. (2003), 'Liver resection (and associated extrahepatic resections) for metastatic well-differentiated endocrine tumors: a 15-year single center prospective study.', *Surgery* **133**(4), 375--382.

6) Eriksson, J.; Stålberg, P.; Nilsson, A.; Krause, J.; Lundberg, C.; Skogseid, B.; Granberg, D.; Eriksson, B.; Akerström, G. & Hellman, P. (2008), 'Surgery and radiofrequency ablation for treatment of liver metastases from midgut and foregut carcinoids and endocrine pancreatic tumors.', *World J Surg* **32**(5), 930--938.

7) Frilling, A.; Li, J.; Malamutmann, E.; Schmid, K.-W.; Bockisch, A. & Broelsch, C. E. (2009), 'Treatment of liver metastases from neuroendocrine tumours in relation to the extent of hepatic disease.', *Br J Surg* **96**(2), 175--184.

8) Gedaly, R.; Daily, M. F.; Davenport, D.; McHugh, P. P.; Koch, A.; Angulo, P. & Hundley, J. C. (2011), 'Liver transplantation for the treatment of liver metastases from neuroendocrine tumors: an analysis of the UNOS database.', *Arch Surg* **146**(8), 953--958.

9) Glazer, E. S.; Tseng, J. F.; Al-Refaie, W.; Solorzano, C. C.; Liu, P.; Willborn, K. A.; Abdalla, E. K.; Vauthey, J.-N. & Curley, S. A. (2010), 'Long-term survival after surgical management of neuroendocrine hepatic metastases.', *HPB (Oxford)* **12**(6), 427--433.

10) Gulec, S. A.; Mountcastle, T. S.; Frey, D.; Cundiff, J. D.; Mathews, E.; Anthony, L.; O'Leary, J. P. & Boudreaux, J. P. (2002), 'Cytoreductive surgery in patients with advanced-stage carcinoid tumors.', *Am Surg* **68**(8), 667--71; discussion 671-2.

11) Hibi, T.; Sano, T.; Sakamoto, Y.; Takahashi, Y.; Uemura, N.; Ojima, H.; Shimada, K. & Kosuge, T. (2007), 'Surgery for hepatic neuroendocrine tumors: a single institutional experience in Japan.', *Jpn J Clin Oncol* **37**(2), 102--107.

12) Jensen, E. H.; Kvols, L.; McLoughlin, J. M.; Lewis, J. M.; Alvarado, M. D.; Yeatman, T.; Malafa, M. & Shibata, D. (2007), 'Biomarkers predict outcomes following cytoreductive surgery for hepatic metastases from functional carcinoid tumors.', *Ann Surg Oncol* **14**(2), 780--785.

13) Kianmanesh, R.; Sauvanet, A.; Hentic, O.; Couvelard, A.; Lévy, P.; Vilgrain, V.; Ruszniewski, P. & Belghiti, J. (2008), 'Two-step surgery for synchronous bilobar liver metastases from digestive endocrine tumors: a safe approach for radical resection.', *Ann Surg* **247**(4), 659--665.

14) Landry, C. S.; Scoggins, C. R.; McMasters, K. M. & Martin, 2nd, R. C. G. (2008), 'Management of hepatic metastasis of gastrointestinal carcinoid tumors.', *J Surg Oncol* **97**(3), 253--258.

15) Lehnert, T. (1998), 'Liver transplantation for metastatic neuroendocrine carcinoma: an analysis of 103 patients.', *Transplantation* **66**(10), 1307--1312.

16) Le Treut, Y. P.; Grégoire, E.; Belghiti, J.; Boillot, O.; Soubrane, O.; Mantion, G.; Cherqui, D.; Castaing, D.; Ruszniewski, P.; Wolf, P.; Paye, F.; Salame, E.; Muscari, F.; Pruvot, F. R. & Baulieux, J. (2008), 'Predictors of long-term survival after liver transplantation for metastatic endocrine tumors: an 85-case French multicentric report.', *Am J Transplant* **8**(6), 1205--1213.

17) Lillegard, J. B.; Fisher, J. E.; Mckenzie, T. J.; Que, F. G.; Farnell, M. B.; Kendrick, M. L.; Donohue, J. H.; Reid-Lombardo, K.; Schaff, H. V.; Connolly, H. M. & Nagorney, D. M. (2011), 'Hepatic resection for the carcinoid syndrome in patients with severe carcinoid heart disease: does valve replacement permit safe hepatic resection?', *J Am Coll Surg* **213**(1), 130--6; discussion 136-8.

18) Máthé, Z.; Tagkalos, E.; Paul, A.; Molmenti, E. P.; Kóbori, L.; Fouzas, I.; Beckebaum, S. & Sotiropoulos, G. C. (2011), 'Liver transplantation for hepatic metastases of neuroendocrine pancreatic tumors: a survival-based analysis.', *Transplantation* **91**(5), 575--582.

19) Mayo, S. C.; de Jong, M. C.; Pulitano, C.; Clary, B. M.; Reddy, S. K.; Gamblin, T. C.; Celinksi, S. A.; Kooby, D. A.; Staley, C. A.; Stokes, J. B.; Chu, C. K.; Ferrero, A.; Schulick, R. D.; Choti, M. A.; Mentha, G.; Strub, J.; Bauer, T. W.; Adams, R. B.; Aldrighetti, L.; Capussotti, L. & Pawlik, T. M. (2010), 'Surgical management of hepatic neuroendocrine tumor metastasis: results from an international multi-institutional analysis.', *Ann Surg Oncol* **17**(12), 3129--3136.

20) Mazzaferro, V.; Pulvirenti, A. & Coppa, J. (2007), 'Neuroendocrine tumors metastatic to the liver: how to select patients for liver transplantation?', *J Hepatol* **47**(4), 460--466.

21) McEntee, G. P.; Nagorney, D. M.; Kvols, L. K.; Moertel, C. G. & Grant, C. S. (1990), 'Cytoreductive hepatic surgery for neuroendocrine tumors.', *Surgery* **108**(6), 1091--1096.

22) Nave, H.; Mössinger, E.; Feist, H.; Lang, H. & Raab, H. (2001), 'Surgery as primary treatment in patients with liver metastases from carcinoid tumors: a retrospective, unicentric study over 13 years.', *Surgery* **129**(2), 170--175.

23) Que, F. G.; Nagorney, D. M.; Batts, K. P.; Linz, L. J. & Kvols, L. K. (1995), 'Hepatic resection for metastatic neuroendocrine carcinomas.', *Am J Surg* **169**(1), 36--42; discussion 42-3.

24) Reddy, S. K.; Barbas, A. S.; Marroquin, C. E.; Morse, M. A.; Kuo, P. C. & Clary, B. M. (2007), 'Resection of noncolorectal nonneuroendocrine liver metastases: a comparative analysis.', *J Am Coll Surg* **204**(3), 372--382.

25) Sarmiento, J. M.; Heywood, G.; Rubin, J.; Ilstrup, D. M.; Nagorney, D. M. & Que, F. G. (2003), 'Surgical treatment of neuroendocrine metastases to the liver: a plea for resection to increase survival.', *J Am Coll Surg* **197**(1), 29--37.

26) Sartori, P.; Mussi, C.; Angelini, C.; Crippa, S.; Caprotti, R. & Uggeri, F. (2005), 'Palliative management strategies of advanced gastrointestinal carcinoid neoplasms.', *Langenbecks Arch Surg* **390**(5), 391--396.

27) Saxena, A.; Chua, T. C.; Sarkar, A.; Chu, F.; Liauw, W.; Zhao, J. & Morris, D. L. (2011), 'Progression and survival results after radical hepatic metastasectomy of indolent advanced neuroendocrine neoplasms (NENs) supports an aggressive surgical approach.', *Surgery* **149**(2), 209--220.

28) Whitney, R.; Tatum, C.; Hahl, M.; Ellis, S.; Scoggins, C. R.; McMasters, K. & Martin, R. C. G. (2011), 'Safety of hepatic resection in metastatic disease to the liver after yttrium-90 therapy.', *J Surg Res* **166**(2), 236--240.
